# Visual exploration patterns of human figures in action: an eye tracker study with art paintings

**DOI:** 10.3389/fpsyg.2015.01636

**Published:** 2015-10-26

**Authors:** Daniela Villani, Francesca Morganti, Pietro Cipresso, Simona Ruggi, Giuseppe Riva, Gabriella Gilli

**Affiliations:** ^1^Dipartimento di Psicologia, Università Cattolica del Sacro CuoreMilano, Italy; ^2^Dipartimento di Scienze Umane e Sociali, Università di BergamoBergamo, Italy; ^3^Applied Technology for Neuro-Psychology Lab, Istituto Auxologico Italiano – Istituto di Ricovero e Cura a Carattere ScientificoMilano, Italy; ^4^Unità di Ricerca in Psicologia dell’Arte, Dipartimento di Psicologia, Università Cattolica del Sacro CuoreMilano, Italy

**Keywords:** face, body parts, visual exploring patterns, complex social scenes, empathic abilities

## Abstract

Art exploration is a complex process conditioned by factors at different levels and includes both basic visual principles and complex cognitive factors. The human figure is considered a critical factor attracting the attention in art painting. Using an eye-tracking methodology, the goal of this study was to explore different elements of the human figure performing an action (face and body parts in action) in complex social scenes characterized by different levels of social interaction between agents depicted in scenes (individual vs. social). The sample included 44 laypersons, and the stimuli consisted of 10 fine art paintings representing the figurative style of classical art. The results revealed different scanning patterns of the human figure elements related to the level of social interaction of agents depicted in the scene. The agents’ face attracted eye movements in social interaction scenes while the agents’ body parts attracted eye movements only when the agents were involved in individual actions. These processes were confirmed specifically in participants with high empathic abilities who became immediately fixated on faces to develop a mimetic engagement with other agents. Future studies integrating other measures would help confirm the results obtained and strengthen their implication for embodiment processes.

## Introduction

Art exploration is a complex process conditioned by factors at different levels, such as basic visual principles and more complex cognitive factors (Quiroga and Pedreira, 2011). Some studies have emphasized the role of “bottom–up” processes, proposing that gaze behavior during picture viewing is affected by physically salient visual features (Berlyne, 1974; Solso, 1994; Ramachandran and Hirstein, 1999; Zeki, 1999; Graham and Field, 2007, 2008; Graham and Redies, 2010). Other studies have recognized the role of “top–down processes,” demonstrating that the goal of visual exploration (task) (Buswell, 1935; Yarbus, 1967; DeAngelus and Pelz, 2009) and the person’s cultural background, art expertise, and familiarity with and interest in a specific work of art (Kristjanson and Antes, 1989; Nodine et al., 1993; Kapoula and Lestocart, 2006; Illes, 2008) are critical elements in influencing art exploration.

Beyond the different factors discussed above, a consensus exists about the role of the human figure as a critical factor in attracting the attention in art painting. In a controlled experimental study with art paintings, Massaro et al. (2012) have recently observed that when represented content included human subjects, content-related top–down processes prevailed over low-level, visually driven, bottom-up processes in guiding the observers’ explorative pattern. This finding indicated that when a human being was portrayed in a painting, the gazing behavior focused mostly on the human figure. The human body has a strong power to orient and attract visual attention at different ages and it plays a fundamental role in esthetic experience (Savazzi et al., 2014).

This behavior is possible because faces and body parts are stimuli of great biological and social significance; they can be rapidly and efficiently processed, and they can engage the attention system (Ro et al., 2007; Crouzet et al., 2010). Several behavioral studies have demonstrated that faces and body parts may have an attentional advantage over other objects (Downing et al., 2001; Ro et al., 2001; Rensink, 2002; Reed et al., 2004).

Why do people instinctively look at a human figure? Up to now, different approaches have been used to answer this question.

In real-life contexts, the ability to derive socially relevant information from faces is a fundamental requirement for normal reciprocal social interaction and interpersonal communication (Pelphrey et al., 2002). This process has been recognized even in the earliest stages of postnatal development (Goren et al., 1975; Johnson et al., 1991), and gaze contact expresses interest or a desire to collaborate (Emery, 2000). Moreover, this instinctive orientation to focus on faces has recently been attributed to both the recognition of emotion (Baron-Cohen et al., 1997) and the similarity of the subjects to the observers themselves (Hari and Kujala, 2009). The human brain has evolved a highly specialized mechanism for facial perception and recognition (Tsao et al., 2006) based on the analysis of its invariant structural features (e.g., the eyes, nose, and mouth). Due to their informative importance, these components remain the preferred attentional targets in healthy adults (Hernandez et al., 2009). One fundamental function of facial processing is to identify individuals, while the other function is to recognize the intentional state of others via the changes in facial features. The second function plays a crucial role in social interactions and constitutes the kernel of “social cognition” (Brothers et al., 1990). Understanding social intention is essentially linked to the brain capacity to recognize the unique morphology of the eye in primates and to provide information regarding the viewing direction of others. According to Langton (2000), not only the face, but also the orientation of head, the posture of the body, and other gestures influence the direction of social attention, and observers process all these cues automatically (Langton and Bruce, 2000).

The importance of the face has been confirmed also in art painting exploration. In fact, several studies have demonstrated that the face is generally the first part of the body that is scanned in portraits and that face-detection process should be particularly sensitive to global cues, mainly the presence of a face contour that activates a configural processing of the stimulus (Young et al., 1987; Abbas and Duchaine, 2008).

Not only the face, but also other body parts attract the viewer’s attention, as pointed out by Massaro et al. (2012) in the observation of art paintings characterized by image dynamism. In static images, the strong attractor was the face, while in dynamic images the attention was equally spread out across various body parts. The authors suggested an embodied explanation of these results: in the first case, embodied simulation of sensations and emotions guided the exploration pattern, while in the second case it was greatly affected by the simulation of actions.

According to the neuroscientific perspective, there is actual substantial evidence of an automatic conversion from vision to action, which occurs even when a person has no intentions to act on the viewed object (Tipper, 2004, 2010; Tipper and Bach, 2008). The automatic conversion from vision to action occurs not only when viewing inanimate objects that can be acted upon, but also when viewing other people’s actions (di Pellegrino et al., 1992). In this sense, the observation of body parts involved in another person’s actions, such as reaching, grasping, and gaze shifts (Grosbras et al., 2005), evokes similar processes in the viewer.

Thus, face and body parts can be seen as elements of the human figure activating different processes: the first related to the understanding of affective states and social intentions and the second related to the automatic activation of a motor-based representation associated with parts of the body.

The situation appears still more complex depending on the social content of the scene. According to Birmingham and colleagues (Birmingham et al., 2008), the social content appears to affect the scanning patterns in complex real-world action scenes. In fact, the level of activity in the scene influences the attention toward the faces when social content is high because face and eye information are critical to understand the social meaning of the action.

To date, this is the first study aiming to investigate how complex social scenes affect the exploration of different parts of the human figure. Specifically, the goal of this study was to investigate the exploration of different elements of the human figure performing an action (face vs. body parts in action) in complex social scenes characterized by the different levels of activity between agents depicted in scenes (individual vs. social) by using an eye-tracking methodology.

We hypothesized (Hp 1) that the orienting of attention toward human figure elements (face and other parts of the body involved in the actions) is different in scenes containing individual actions (several agents performing something separately) compared to scenes containing social actions (several agents performing something together). Specifically, we expected individuals to pay a greater attention to faces rather than arms in social action condition compared to individual action condition. In individual action condition individuals are expected to look at agents’ parts of the body involved in the action to understand the nature of the action, while in social action condition individuals are expected to look at agents’ faces to understand the social nature of the interaction between the agents.

To verify this hypothesis we used an eye-tracking methodology to explore human figure elements in complex social scenes depicted in art paintings. Eye tracking is a common methodology used to trace the inner operations of attention and cognition (Just and Carpenter, 1976; Jacob and Karn, 2003; Rayner, 2009) and to understand art exploration. Tracking eye movements is advantageous because they are fast and natural (Jacob and Karn, 2003), and it is possible to identify both specific areas of interest (salient regions of an image), as well as specific viewer’s explorative patterns.

Furthermore, we considered that the observation of body in action and motor resonance derived from simulation processes has been identified as a foundation of empathy (Jola et al., 2012). Freedberg and Gallese (2007) indicated that when contemplating artistic works by virtue of their visual content, it is possible to live embodied experiences and feel an empathetic engagement with the work of art. These feelings might consist of the viewer’s perceived understanding of the emotions of the agents represented in the pictures or, most strikingly, a sense of inward imitation of their observed actions, resulting in a seemingly mimetic engagement with the human figures depicted in the paintings.

Thus, we wondered if the observation of the body in action of the depicted agents might vary among people according to their empathic dispositions.

Starting from these premises, we aimed to examine (Hp 2) whether the observer’s individual characteristics in terms of emphatic abilities were associated with a specific scanning of the human figure (face and other parts of the body involved in actions) in scenes containing individual or social actions. For this purpose, we introduced a self-report instrument to assess the empathic responsiveness of participants and to understand whether this ability could affect the exploration of the individual and social scenes within an art painting.

## Materials and Methods

### Participants

Because non-expert viewers are more likely to observe human features compared to expert viewers (Vogt, 1999; Vogt and Magnussen, 2007), we selected a non-expert sample. The laypersons’ group (*n* = 44) consisted of university students or volunteers who did not major in art but had taken a college art course. Of these participants, 23 were female, 21 were male, and the mean age was 27.7 years (range 20–48 years; *SD =* 6.62). All participants had normal or corrected-to-normal vision and signed an informed consent form prior to the experiment. The study was approved by the Local Ethics Committee of the Psychology Department of Catholic University of Sacred Heart of Milan and was performed in the Laboratory of Communication Psychology at the same University.

### Stimuli

The stimuli consisted of 10 fine art paintings by both renowned and unknown artists, which represented the figurative style of classical art from the 17th to 19th centuries. The paintings, which were downloaded from various digital art libraries, were divided into two categories that represented agents engaged in bodily actions characterized by two levels of social interaction: individual and social actions. The two categories are descripted in **Table 1**.

**Table 1:** Stimuli categorization.

*Individual actions*	In the same scene, several agents were present but were not interacting. At least one of the agents was performing a bodily action (five stimuli).

*Social actions*	In the same scene, several agents were present and interacting by accomplishing complementary actions; that is, one’s action corresponded with another’s reaction (e.g., one agent pulls and the other pushes) or one performed an action on the other’s body while the other watched (e.g., the first agent is looking at the second who is touching the first; five stimuli).

To confirm the stimuli categorization (individual vs. social actions scenes), 12 non-expert judges who were not art scholars, architects, or painters (six females, six males; range 19–60 years) selected the stimuli. The judges were given descriptions of each of the two categories and were asked to categorize 78 paintings containing human figures and representing the aforementioned style. The agreement index (Cohen’s Kappa coefficient >0.60, *p* < 0.005) between non-expert judges was used to select 10 paintings for the experimental phase. The Supplementary material contains a table that lists each painting’s title, date, corresponding category, and digital resolution.

Although the literature shows that gazing focuses on the human figure in paintings (Massaro et al., 2012), before doing the experiment, we controlled the bottom–up processes using the visual saliency model (Itti and Koch, 2000). This model comprises a number of parallel channels for processing different feature types, such as luminance, orientation, and color, and the outputs from each channel are combined to produce a single, feature-independent salience map. This salience map signals salient or “interesting” locations in the visual scene, regardless of which features contributed to the salience. It has been shown that such salience maps can predict locations likely to be fixated by human observers with accuracy significantly better than chance (Parkhurst et al., 2002). Furthermore, Fuchs et al. (2011) investigated the predictive value of Itti and Koch’s salience model (Itti and Koch, 2000, 2001) on gaze behavior for photographs and paintings and suggested that the salience map works well also for artworks.

We used the result of the visual saliency analysis to confirm that the human figure could be regarded as an interesting position in the selected paintings.

**FIGURE 1:**
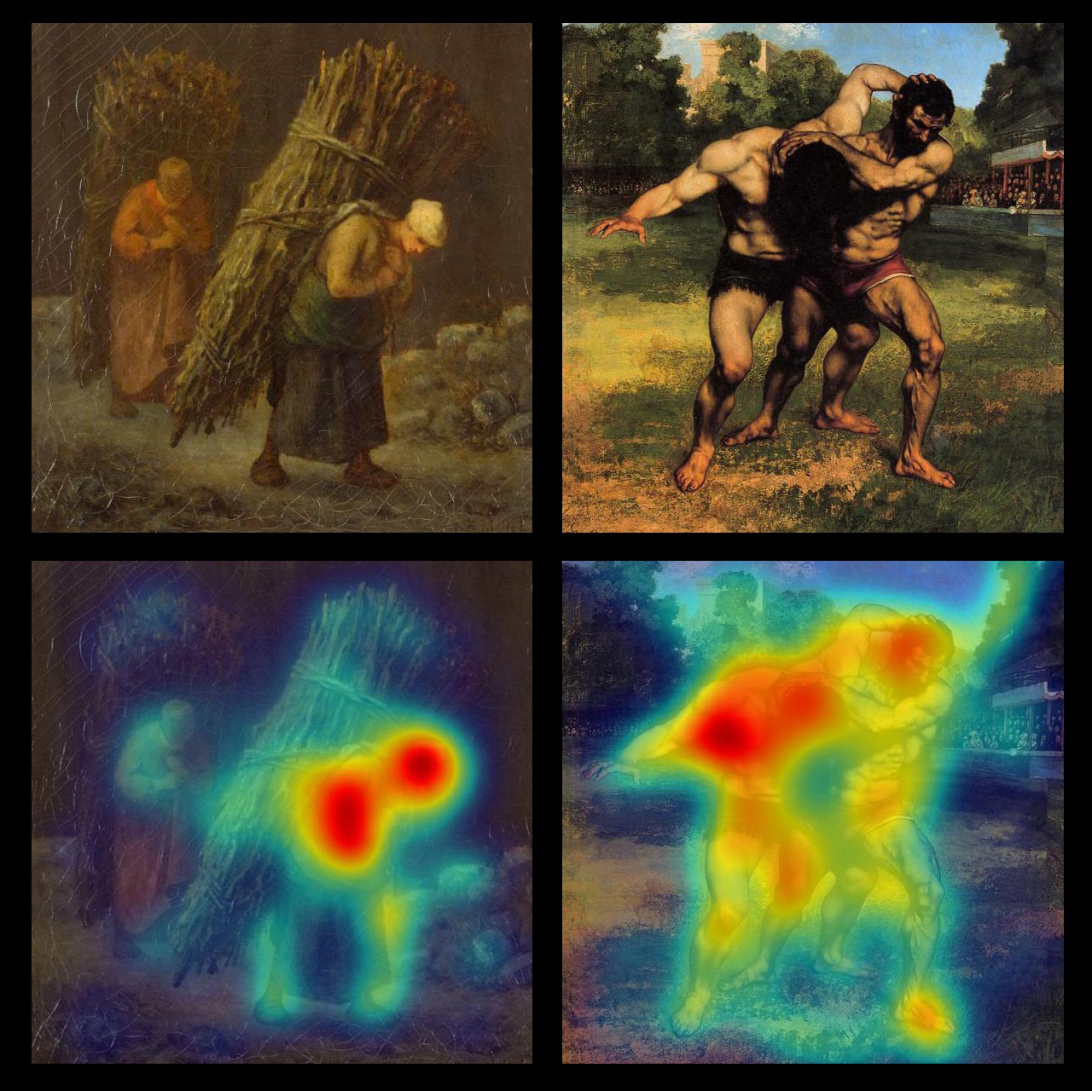
**Representative paintings for each category. (Top)** On the left, Individual Action (*Peasant Women with Brushwood* by Jean François Millet, 1858). **(Top)** On the right, Social Action (*The Wrestlers* by Gustave Courbet, 1853). **(Bottom)** Left and right: paintings with visual saliency map.

**Figure 1** displays a representative painting for each category and the result of the visual saliency analysis. It shows that both human figure elements (such as the face and other parts of the body involved in the actions) are considered salient or “interesting” locations in the visual scene.

Because evidence suggests that presenting titles together with artworks may affect how viewers process the artworks (Leder et al., 2006), we displayed the paintings without titles to avoid any effect of the titles on the viewers’ exploration, understanding, and evaluation of the paintings.

### Measurements

#### Eye-tracking Data Acquisition

Tobii Eye-Tracker X120, a stand-alone eye-tracking unit that uses an infra-red system to sample eye position by capturing pupillary and corneal reflections every 1/120 of a second, was used to record the participants’ eye positions. The system is accurate within 0.5°. The tracker was set on the desk between the subject and monitor, and the viewing distance was approximately 60 cm from the computer screen. The visual angle covered by the paintings measured on average 20° (both on horizontal and vertical axes) so that stimuli were presented within the 30° of focal visual field and participants could freely move their eyes without turning their head.

Software for the eye tracker processes the eye-movement indicators in terms of number and duration of fixations. Among the indicators, we focused on *fixations*, which referred to the positioning of a target feature of interest on the fovea for a variable period (approximately 300 ms per fixation). Max angle between fixations was 0.5° and max time between fixations was 75 ms. Minimum fixation duration was established at 60 ms. Specifically, the *fixations duration* refers to the amount of time in seconds that the fovea is directed at a specific Region of Interest (ROI), such as salient regions of an image, while *fixations before* refers to the number of fixations that occurred before the viewer examined the ROI. According to the model described by Shimojo et al. (2003), we considered *fixations before* and *fixations duration* to be indicators of interest in and preference for the object of fixation, respectively.

The categories that were found to draw attention to specific ROIs related to the human figure, drawn manually, were *faces*, which are the part of the human figure that typically attracts the viewer’s eye, and *arms*, which in our scenes, are the body parts involved in a bodily action movement (e.g., subjects were likely to pick up or handle something using their arms) that could lead to a mimetic engagement with the human figures depicted in the paintings. All paintings presented in the experiment included at least two but not more than four human figures. To perform a comparison between fixations toward faces and arms, we balanced the selected ROIs containing faces and those containing arms so that they covered no more than 5% of the entire scene.

#### Interpersonal Reactivity Index (IRI)

To evaluate the participants’ reactions and ability to derive socially relevant information, we used the most widely used instrument to assess empathic responsiveness, the Interpersonal Reactivity Index (IRI; Davis, 1980). The IRI comprises 28 items measuring four dimensions, *Empathic Concern* (feeling emotional concern for others), *Perspective Taking* (cognitively taking another’s perspective), *Fantasy* (emotional identification with fictional characters), and *Personal Distress* (negative feelings in response to the distress of others).

Each of these dimensions consists of seven items measured on a five-point Likert ranging from 1 (“does not describe me well”) to 5 (“describes me very well”). The Italian validated version of the questionnaire (Albiero et al., 2006), which was used in this experimental paradigm, demonstrated satisfactory reliability and good internal consistency (*Empathic Concern* α = 0.61; *Perspective Taking* α = 0.64; *Fantasy* α = 0.74, *Personal Distress* α = 0.64).

### Design and Procedure

According to Locher et al. (2007), 10 s is a sufficient time to obtain an overview of a picture while 30 s is the average period of observation for an esthetic judgment when unlimited time is given, as in the case of real art museum visitors (Smith and Smith, 2001). For this reason, we defined a presentation time of 10 s for each painting.

Participants were first asked to fill out the questionnaire and to subsequently perform the experiment, which consisted of a viewing sequence of all the paintings in a randomized order. To investigate the exploration of human figure elements, the participants were instructed to look at the human figures represented in each painting. Even if the visual saliency model confirmed that the human figure could be regarded as an interesting position in the selected paintings, a follow up study has been carried out to verify that the instruction to look at the human figures did not bias participants’ exploration patterns.

The software ran on a stimulus computer connected to the eye-tracking computer to provide correct timing. An initial calibration pattern was displayed to the participants before running the eye-tracker session. After showing each painting, the participants were given 15 s to orally answer one question while maintaining their gaze on the screen. According to the procedure followed by Pihko et al. (2011), we asked “Have you seen this painting before?” This yes or no question was used to determine the participant’s previous knowledge of the painting. On the average, each participant was only familiar with one of the paintings. The known painting differed across participants and for this reason; it was not excluded from the analysis.

**FIGURE 2:**
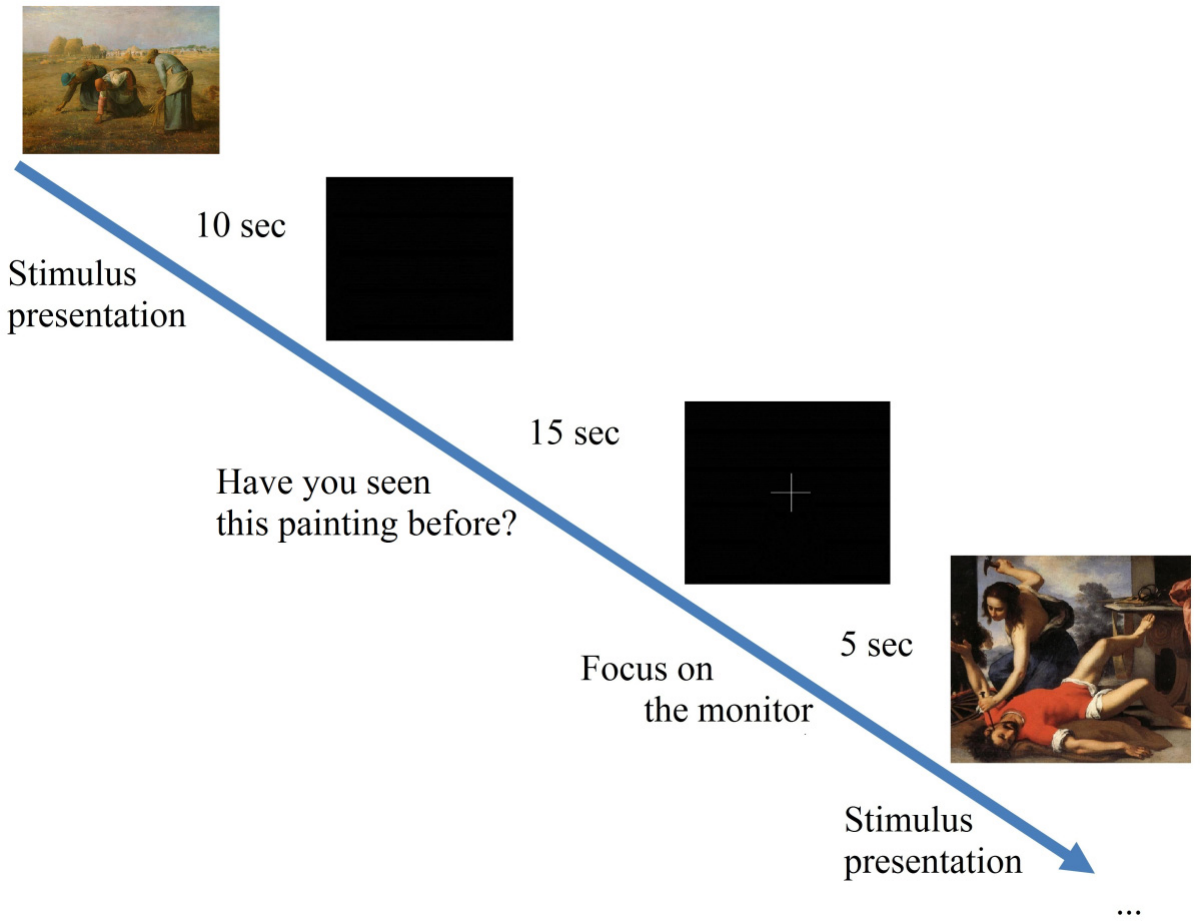
**Experimental procedure**.

Finally, a cross on the screen indicated the end of the answering period and the appearance of the stimulus. The experimental procedure is depicted in **Figure 2**. The full experiment, including the preparation time, lasted approximately 30 min per participant.

## Results

The data analysis related to eye movement indicators and the self-report questionnaire was performed using SPSS analysis software (version 19.0; Statistical Package for the Social Sciences, Chicago, IL, USA).

### Visual Scanning Patterns of Human Figure Elements in Action Scenes with Different Levels of Social Interaction

Hypothesis 1 stated that the human figure in art paintings characterized by action representation is scanned differently in scenes containing individual actions compared to scenes with social actions. To test this hypothesis we performed a two-way Repeated Measures ANOVA with body parts (face vs. arms) as one factor and social interaction (individual vs. social actions) as another factor. Descriptive data are reported in **Table 2**.

**Table 2:** Descriptive statistics of the two factors: body parts and social interaction.

	Measures [mean (*SD*)]	
	Fixations_before	Fixations_duration
Faces	6.334 (0.722)	1.792 (0.158)
Arms	5.227 (0.387)	1.440 (0.089)
Individual actions	5.939 (0.501)	1.453 (0.078)
Social actions	5.623 (0.417)	1.779 (0.105)

Concerning the scanning of individual vs. social actions scenes, we found a significant main effect in terms of fixations duration [*F*(1,44) = 28.897, *p* < 0.001, $\eta_{p}^{2}$ = 0.402]. The effect of fixations before was non-significant [*F*(1,44) = 0.451, *p* = 0.505, $\eta_{p}^{2}$ = 0.010]. This finding showed that participants took more time to observe face and arms in social scenes than in individual scenes while no differences were found about how many eye fixations were logged before participants spotted the ROIs of faces and arms.

Concerning the scanning of body parts, we found no significant main differences between faces and arms in terms of fixations before [*F*(1,44) = 1.718, *p* = 0.197, $\eta_{p}^{2}$ = 0.038] and fixations duration [*F*(1,44) = 3.538, *p* = 0.067, $\eta_{p}^{2}$ = 0.076]. This finding indicated that the participants looked similarly at the ROIs of faces and arms of human agents performing actions, regardless of the presence, or absence of social interaction.

The primary purpose of two-way repeated measures ANOVA is to understand whether there is an interaction between these two factors on the dependent variable. In this case, we found a significant interaction effect between body parts and social activity both in terms of fixations before [*F*(1,44) = 32.563, *p* < 0.001, $\eta_{p}^{2}$ = 0.431] and fixations duration [*F*(1,44) = 50.368, *p* < 0.001, $\eta_{p}^{2}$ = 0.539].

**FIGURE 3:**
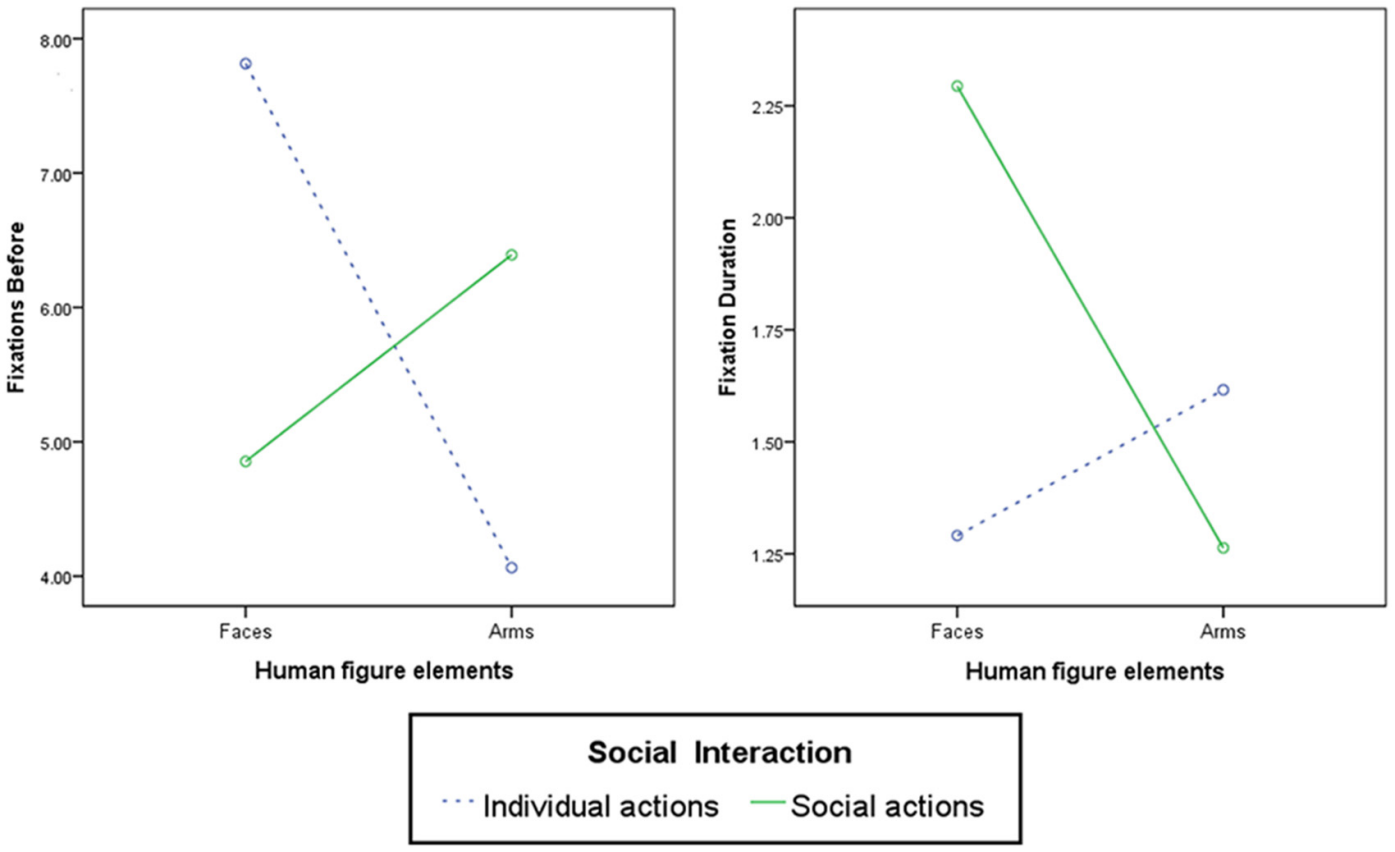
**Statistical interaction effects between human figure elements (faces vs. arms) and social interaction levels (individual vs. social actions; Fixations before and Fixations duration)**.

**Figure 3** may help us interpret this important result. When the scenes depicted individual actions, the participants showed a higher number of fixations before examining the ROIs of faces and a lower number of fixations before examining the ROIs of arms. When the depicted scenes were social actions, the participants showed a higher number of fixations before examining the ROIs of arms and a lower number of fixations before examining the ROIs of faces. This finding indicated that the participants looked immediately at the arms in case of individual actions and looked immediately at the faces in case of social actions. The same result was obtained in terms of the fixations duration. When the depicted scenes were individual actions, the participants exhibited longer fixation duration toward the ROIs of arms and shorter duration toward the ROIs of faces. When the depicted scenes were social actions, the participants showed longer fixations duration toward the ROIs of faces and shorter duration toward the ROIs of arms. This finding indicated that the participants maintained their fixations on the arms in case of individual actions and on faces in case of social actions.

### The Role of Individual Empathic Abilities on Human Figure Scanning

According to hypothesis 2, we investigated the role of empathy, which was measured by using the IRI questionnaire (Davis, 1980; Albiero et al., 2006) to orient visual scanning of the human figure in art paintings with different levels of social interaction. To answer this question, we performed a series of linear regression analysis with each IRI factor (*Empathic Concern, Perspective Taking, Fantasy and Personal Distress*) as independent variable and eye movements toward the ROIs of faces and arms as dependent variables. A Bonferroni correction has been applied for multiple comparisons.

Our results showed that in social actions scenes *Empathic Concern* had a significant effect on the number of fixations that occurred before examining the faces, while *Perspective Taking* had a significant effect on the number of fixations that occurred before examining the arms, as shown in **Table 3**.

**Table 3:** Model summary of linear regression between empathic concern and perspective taking (Interpersonal Reactivity Index, IRI) and paintings categories in terms of fixations before.

Category	Dependent variable	Independent variable	*R*	Adjusted *R*^2^	*F*(1,43)	Significance	β	*SE*
*Social actions*	Faces	Empathic concern	0.482	0.214	12.724	0.002	-5.126	1.437
	Arms	Perspective taking	0.365	0.133	6.453	0.030	2.644	1.041

In the most complex social category, such as that involving social action, participants that feel emotional concern for others showed fewer fixations before looking at the faces. Participants able to cognitively taking another’s perspective showed fewer fixations before looking at the arms.

Furthermore in individual actions our results showed that *Empathic Concern* had a significant effect on the number of fixations before looking at the arms (*R* = 0.344, Adjusted *R*^2^ = 0.097, *F* = 65.628, *p* = 0.044, β= –1.870, *SE* = 0.788). No significant effects have been found for other IRI factors (*Fantasy* and *Personal Distress*) and no significant effects were found related to the other eye movement indicator (fixations duration), which demonstrated that empathic abilities have an effect only on orienting the visual scanning of human figure elements.

### Follow Up Study

Since the found results might be biased by the instruction to look at the human figures, another experiment was carried out with a new sample of participants that were not instructed to look at the human figures represented in each painting and that were not asked to fill out the IRI questionnaire.

The goal of this follow up study was to compare the visual exploration patterns of the two groups (with and without instruction to look at the human figures) toward the different body parts in individual and social action scenes in order to confirm results obtained with the main study. The new laypersons’ group (group 2, *n* = 15) consisted of university students or volunteers who were not art majors, but had taken a college art course. Of these participants, 9 were female, 6 were male, and the mean age was 30.1 years (range 22–50 years; *SD* = 8.63).

We performed a 2 × 2 × 2 mixed-design ANOVA, with body parts (face vs. arms) and social interaction (individual vs. social actions) as within-subjects factors, and instruction (group with vs. group without instruction to look at the human figures) as a between-subjects factor. Results revealed no significant interaction effects between body parts and instruction groups both in terms of fixation duration [*F*(1,57) = 0.641, *p* = 0.427, $\eta_{p}^{2}$ = 0.011] and fixations before [*F*(1,57) = 3.711, *p* = 0.059, $\eta_{p}^{2}$ = 0.061]. Furthermore, no significant interaction effects have been found between social interaction levels and instruction groups both in terms of fixation duration [*F*(1,57) = 0.018, *p* = 0.893, $\eta_{p}^{2}$ = 0.00] and fixations before [*F*(1,57) = 0.882, *p* = 0.352, $\eta_{p}^{2}$ = 0.015].

Thus, the follow up study confirm that participants instructed to freely look at the paintings showed equivalent visual exploration patterns to participants instructed to look at the human figures represented in each painting. Moreover, filling in the questionnaire before the experiment did not influence the participants’ visual exploration patterns.

Descriptive statistics are presented in **Table 4**.

**Table 4:** Descriptive statistics of eye movements of the two groups.

Category	Region of Interest (ROIs)	Eye movements	Mean (*SD*)
Individual actions	Faces	Fix_duration	Group 1: 1.28 (0.91) Group 2: 1.34 (0.80)
		Fix_before	Group 1: 7.91 (5.90) Group 2: 6.28 (5.00)
	Arms	Fix_duration	Group 1: 1.62 (0.68) Group 2: 1.18 (0.60)
		Fix_before	Group 1: 4.00 (2.33) Group 2: 6.35 (4.67)
Social actions	Faces	Fix_duration	Group 1: 2.32 (1.35) Group 2: 2.13 (1.39)
		Fix_before	Group 1: 4.90 (4.61) Group 2: 4.97 (6.21)
	Arms	Fix_duration	Group 1: 1.15 (0.56) Group 2: 0.91 (0.54)
		Fix_before	Group 1: 6.56 (4.23) Group 2: 8.87 (2.63)

## Discussion and Conclusion

As the human figure attracts gazing behavior in paintings, the goal of the study was to use eye-tracking methodology to investigate, with untrained participants, which part of a human figure orients visual exploration. For this reason, we analyzed selected paintings containing complex social scenes (Kuchincke et al., 2009; Graham and Redies, 2010), which are characterized by body dynamism and the presence/absence of the agents’ social interaction.

The first hypothesis, which stated that the human figure in art paintings characterized by action representation is scanned differently in scenes containing individual actions compared to scenes with social actions, was confirmed. The primary purpose of the analysis was to understand whether there was an interaction between the two factors (body parts and activity) on visual exploration patterns. In our analysis, we found significant interaction effects of scene characteristics in terms of social interaction among agents and human figure elements on the participant’s fixations. In paintings representing social actions, faces gained the traditional attractiveness, probably associated with the attempt to recognize intentions and emotions from the face (Baron-Cohen et al., 1997; Wallraven et al., 2009), and participants looked first at the ROIs of faces and for a longer period. The opposite pattern was found in paintings representing individual actions, where the participants looked first at the ROIs of arms and for a longer period.

This result was similar to that found by Birmingham et al. (2008). When the social content of a scene was high, that is, when several agents are performing something together, the social action drew the participants’ attention toward the face because facial information is critical to understand the social meaning of the action. However, when the social content of a scene is low, that is, when several agents are performing something separately, the individual action draws attention away from the face because face information is not critical to understand the action.

Moreover, this result was consistent with the embodied perspective proposed by Freedberg and Gallese (2007), despite some slight differences. In the observation of still images of actions in works of art, the authors referred to a motor simulation process in which the physical response can be located in the part of the body engaged in purposeful physical actions. From our results, we found that in experiencing an artwork, the observers were not generally focused on the entire human figure; instead, they appeared to focus on different human figure elements depending on the feature of the scene in terms of social interaction. Moreover, the body parts involved in the action attracted the viewers’ attention when the represented action was individual rather than social. Thus, it was likely that the viewers looked at arms to convert information from visual perception into corresponding physical movements and to understand the effort required to perform that action. In contrast, when the represented action was social, the body parts involved in the action were not highly attractive. In this case, the viewers focused on faces of agents involved in the action most likely to understand the social nature of the interaction between the agents.

According to Tessari et al. (2012), the ability to detect the intentions and the emotions of others during an interaction requires individuals to anticipate the consequences of their behavior. This was primarily possible via the observation of the agents’ body postures because intention could be automatically extrapolated from the body movement based on a shared motor representation. From our study, the face appeared to constitute socially relevant information in terms of valence and potential action when the intention has a social nature while body movement appears to be a determining element only when the movement presents a non-social nature.

This perspective appears to be critical to understand the visual exploration of figurative art paintings, particularly when the viewers are laypersons and the representations include several agents engaged in bodily actions.

This study also aimed to examine (hypothesis 2) whether the observers’ individual characteristics in terms of emphatic abilities resulted in the differential scanning of the human figure (face and other body parts involved in the actions) in scenes containing individual actions compared to scenes with social actions. Other studies have recently showed that among the individual characteristics of the viewers, gender and personality emerged as critical aspects that differentially oriented attention in visual exploration based on the fact that what we look helps construct what we see (Mercer et al., 2012). In this study, the empathic level of the participants affected the initial orientation and screening, but not further evaluation (Russo and Leclerc, 1994). Our results suggested two different roles of affective and cognitive empathic abilities in scenes with high level of social interaction. On the one hand, participants with higher levels of empathic concern (i.e., participants’ emotional identification with agents depicted in the scene) showed fewer fixations before focusing on faces. This finding is consistent with the interpretation that participants were paying attention to the emotional content of the scene – agents’ faces – in order to understand others and simulate their emotional state (Vuilleumier and Driver, 2007). It further suggests that emotional information processing is related to self-report of affective empathy (Hooker et al., 2010). On the other hand, participants’ ability to cognitively adopt another’s perspective and understand the meaning of the represented action resulted in the attraction toward the body parts involved in the action. This result is consistent with the notion that cognitive empathy skills, such as perspective-taking, relies on the use of internal models of observed action in order to understand another person’s situation (Gallese, 2007). These processes should be further investigated to confirm the different role of affective and cognitive empathy in orienting of attention toward human figure elements.

Taken together, the results of the present study revealed a critical role of social content in the exploration of the human figure elements in action. As demonstrated by Bayliss and Tipper (2005), it is important to investigate the role of the context in which orienting of attention occurs. This aspect is particularly important in investigating the attentional control of social orienting (Jordan and Tipper, 1998).

The face is confirmed to be as one of the most important elements of the human figure in attracting the viewers’ attention. Through facial observation, the participants understand others and simulate the emotional state via the generation of representations of the associated body state both in case of individual and social actions (Adolphs, 2002; Gallese et al., 2004). This process was confirmed specifically in participants with high empathic abilities that immediately orient their fixations on faces.

We must consider that the non-homogeneity in the visibility of the faces is a weakness of the study, and future studies are encouraged to include stimuli in which the faces are visible and detailed. Nevertheless, we want to emphasize that we used a configural or holistic approach to face processing that does not consider agent facial features (Penton-Voak et al., 2006). Specifically, we focused on orienting of attention toward different parts of human figure (Frischen et al., 2007). Emotional signals may still be perceived under difficult circumstances, such as when faces are behind a veil (Fischer et al., 2012). In this sense, the visibility or orientation of the faces did not compromise the results of the study, which can be generalized to other situations where expressive cues are not visible. Furthermore, future studies are encouraged to include motor actions where other body parts are involved in the bodily action movement.

Finally, as this study included a single experiment, this limitation calls for viewing the obtained results with caution. Thus, additional studies are required to investigate affective and cognitive processes by integrating other measures, such as psycho-physiological ones, which are more suitable to understand the activation of the mimetic engagement with other agents represented in the pictures and their implication for embodiment processes.

## Conflict of Interest Statement

The authors declare that the research was conducted in the absence of any commercial or financial relationships that could be construed as a potential conflict of interest.
