# Supplementary material for: Visual exploration patterns of human figures in action: an eye tracker study with art paintings
Source: Front Psychol. 2015 Oct 26;6:1636. doi: 10.3389/fpsyg.2015.01636 (PMC4620395; doi:10.3389/fpsyg.2015.01636)
Supplement: Supplementary file 1 [file Data_Sheet_1.DOCX]

**Supplementary material**

Paintings included in the two categories

| **Category** | **Artist** | **Title** | **Date** | **Location** | **Image size in pixels** |
| --- | --- | --- | --- | --- | --- |
| Individual Actions | Jean-Francois Millet | *Peasant Women with Brushwood* | 1858 | Hermitage Museum, Amsterdam | 600×777 |
|  | Jean-Francois Millet | *The Gleaners* | 1857 | Orsay Museum, Paris | 797×600 |
|  | Gustave Courbet | *The Wheat Sifters* | 1854 | Nantes Fine Art Museum, France | 760×600 |
|  | Gustave Courbet | *The Stone Breakers* | 1849 | Gallery Neue Meister, Dresden, Germany | 800×487 |
|  | Jean François Millet | *Trussing Hay* | 1850 | Louvre Museum, Paris | 800×589 |
| Social Actions | Caravaggio | *Sacrifice of Isaac* | 1603 | Uffizi Gallery, Florence | 769×600 |
|  | Jean-Honore' Fragonard | *The Lock* | 1780 | Louvre Museum, Paris | 638×783 |
|  | Felice Ficherelli | *Jael and Sisera* | 1616 | Uffizi Gallery, Florence | 800×627 |
|  | Pierre-Auguste Renoir | *Dance at Bougival* | 1883 | Museum of Fine Arts, Boston | 417×800 |
|  | Gustave Courbet | *The Wrestlers* | 1853 | Museum of Fine Arts, Budapest, Hungary | 600×796 |
